# Surface-Token-Guided Query Refinement for Weakly Supervised RGB Apple Defect Sensing and Visible Surface-Condition Scoring

**DOI:** 10.3390/s26206536

**Published:** 2026-10-15

**Authors:** Zengyu Wang, Yasenjiang Musha

**Affiliations:** School of Mechanical Engineering, Xinjiang University, Urumqi 830000, China; wangzengxju@163.com

**Keywords:** RGB vision sensor, apple defect sensing, weak supervision, detection transformer, visible surface condition, synthetic-to-real transfer, postharvest inspection

## Abstract

Low-cost RGB imaging is attractive for postharvest apple grading, yet an image-level defect label does not reveal where damage occurs or how much of the visible surface it affects. This study examines whether folder-labeled images can support defect recognition, weak localization, binary health assessment, and surface-condition scoring within one model. The resulting framework, WS-DA-DETR, retains DETR set prediction but conditions its queries on local texture residuals, boundaries, and color deviations extracted from the apple surface. Appearance-derived masks and boxes provide a conservative localization curriculum, while auxiliary contrastive and cross-head constraints relate spatial predictions to image-level condition. Manual spatial annotations are reserved for held-out evaluation. Experiments used 10,000 real and synthetic apple images in exact-duplicate-grouped mixed-domain and cross-domain protocols. The mixed-domain test yielded 89.5% four-class accuracy and 96.3% health AUC. On a held-out manually annotated subset, ${\mathrm{AP}}_{50}$ was 54.6%, ${\mathrm{AP}}_{50:95}$ was 32.3%, mask IoU was 48.7%, and localization recall at IoU 0.5 was 74.5%. The Pearson and Spearman correlations with human surface-condition ratings were 0.68 and 0.66, respectively. Model-only inference required 30.2 ms per image (33.1 FPS) on an RTX 3090 GPU (NVIDIA Corporation, Santa Clara, CA, USA). These results support a proof of concept on centered, single-apple images, using folder labels and appearance-derived spatial targets for training and a separate 200-image manual subset for evaluation.

## 1. Introduction

Visible damage affects postharvest grading, storage decisions, market value, and waste. Human inspection recognizes many obvious defects but depends on consistent judgments of type and visible extent. Camera-based inspection provides a repeatable record, yet apple surfaces are difficult to interpret: bruises may be diffuse and low contrast, scab lesions are often small and irregular, rot can cover a broad region, and highlights or the stem and calyx may resemble damage.

RGB cameras are convenient where a compact sensor must be fitted to a sorting line. They require less specialized hardware than multispectral systems, but this simplicity comes with limited spectral discrimination and greater sensitivity to illumination and color variation. Earlier machine-vision systems addressed these difficulties through controlled lighting, hand-crafted color or texture descriptors, and explicit rejection of the stem and calyx [1,2,3]. Multispectral acquisition can make subtle surface changes easier to separate, although specialized cameras, illumination, and calibration add complexity [4,5,6]. RGB inspection is useful not because every defect is conspicuous in three channels, but because it provides a practical setting in which spatial evidence can be examined.

The choice of supervision determines what such a system can report. Convolutional networks trained on real and virtual fruit images have produced strong image-level recognition results [7], and related work has considered pixel-level segmentation [6], complex-scene detection [8], and multispectral defect extraction [5]. A classifier needs relatively inexpensive category labels, yet it cannot identify the surface region responsible for its decision or estimate the visible extent of damage. Detectors and segmenters provide this spatial information, but their boxes and masks require substantially more annotation. That supervision is absent from many public fruit-image archives, including the folder-labeled data considered here.

DETR offers a direct set-prediction formulation based on an encoder–decoder and learned object queries [9]. Its descendants improve multi-scale sampling, convergence, query initialization, and efficiency [10,11,12]. Those advances do not by themselves remove the need for spatial targets. In addition, a fixed query bank and global token interaction provide no explicit preference for a faint bruise or a small scab region over healthy peel and background. Weakly supervised object localization instead derives spatial evidence from image-level labels. CAM and Grad-CAM are common choices [13,14], but a classification objective often concentrates on the most discriminative patch rather than the full lesion. Transformer-based localization can use long-range context, while continuity of local evidence remains difficult [15]. For apple inspection, the relevant question is thus not simply whether a heatmap can be produced, but whether its evidence can be linked to defect type and surface condition and then evaluated against annotations that were not used to create the weak training cue.

This study examines whether folder-level RGB apple images can support four related outputs: defect class, weak lesion localization, binary health status, and a continuous visible surface-condition score. WS-DA-DETR keeps DETR’s set-prediction structure while making two connected changes to how local surface evidence enters the model. Texture, boundary, and color-deviation cues are first incorporated into the highest-resolution feature map; the resulting surface representation then adapts the query bank to each image. Pseudo regions supply a conservative training curriculum, whereas manual boxes and masks are used only to assess localization on held-out images.

The contributions are organized around the sensing task rather than individual training components:

- The folder-labeled task is formulated to produce image-level class and health predictions together with weak box and mask localization and a visible surface-condition score. Human lesion annotations remain separate from model training and are reserved for evaluation.
- A surface-aware query pathway connects local appearance deviations to DETR set prediction. Surface defect token encoding (SDTE) incorporates feature-space texture residuals, Sobel boundaries, and local color deviations, and defect-guided query refinement (DGQR) uses the resulting evidence to adapt the query bank for each image.
- The evidence is examined at several levels through exact-duplicate-grouped mixed-domain testing, two cross-domain directions, a manually annotated localization subset, a direct pseudo-target audit, factorial component ablation followed by auxiliary-loss analysis, and batch-one model timing. These protocols distinguish image recognition from spatial accuracy, supervision quality from learned-model performance, and transfer between synthetic and real images.

## 2. Related Work

### 2.1. Apple Defect Detection and Optical Sensing

Classical apple-inspection pipelines separate fruit from the background, correct uneven illumination, and distinguish candidate blemishes from the stem and calyx using color or texture descriptors and shallow classifiers [2,3]. Each threshold has an interpretable role, but changes in illumination, cultivar color, or camera settings can alter the cues on which the decision depends.

Deep segmentation reduces hand-selected feature engineering and has been applied to visible, near-infrared, and multi-band fruit imagery [5,6]. It estimates lesion shape and extent, but requires pixel-wise annotation; multi-band acquisition also assumes additional hardware. Detection studies learn boxes in more complex scenes [8], yet likewise start from explicit spatial labels. These methods inform the desired output but do not directly fit a folder-labeled dataset.

The Healthy-Defective-Fruits dataset contains real and synthetic apple and mango images and was studied as an image-recognition problem; MobileNetV2 was effective under that formulation [7,16]. Its local apple subset provides fresh, bruise, rot, and scab folders in both domains. These labels support category learning but cannot establish whether a prediction is based on a lesion, background, illumination, or a synthetic-generation cue. Weak training cues and manually assessed localization must therefore remain separate.

### 2.2. Transformers for Detection and Weak Localization

Vision architectures balance local detail, scale, and long-range interaction differently. ViT applies global self-attention to patches [17]; Swin Transformer builds a hierarchy with shifted windows [18]; and ConvNeXt retains convolutional locality in pyramid features [19]. None alone guarantees that a small or diffuse surface defect will dominate. Fine spatial cues must be preserved while evidence from separate regions interacts.

DETR casts detection as set prediction with learned queries, removing hand-designed anchors and non-maximum suppression [9]. Deformable DETR, DINO, and RT-DETR improve multi-scale attention, optimization, query initialization, or efficiency [10,11,12]. Standard Hungarian matching still assumes ground-truth boxes. With class folders alone, the detector requires another spatial cue, and queries shared across images do not know where a surface deviation occurs.

WSOL derives guidance from discriminative features or attention rather than box annotation. CAM and Grad-CAM may cover only the patch needed for classification [13,14]. Affinity propagation and transformation-consistency constraints provide additional ways to expand or stabilize weak activation maps [20,21]. LCTR addresses local continuity [15]; other work considers category-aware evidence or generative prompts [22,23], frozen vision–language representations [24], and cross-domain proposal learning [25]. Localization quality also depends on score-map thresholds, validation supervision, and model-selection protocols [26,27]. Mixed supervision therefore uses weak cues to reduce, rather than erase, dense annotation needs [28]. Here, appearance-derived regions guide training and manual labels independently test localization. Related transformer approaches include WFS-DETR, which transfers object-localization and integrity information to novel categories using image labels [29], and Omni-DETR, which incorporates unlabeled, weakly labeled, and fully labeled examples through a teacher–student detector [30]. Their pretraining and annotation mixtures differ from the present fixed-category apple task and require explicit supervision accounting in a controlled comparison.

Vision–language and open-vocabulary segmentation provide a complementary route when target categories or boundaries are not fixed in advance. Frozen CLIP representations can support weakly supervised semantic segmentation [24], while Zhao et al. use VLM-derived semantic features to prompt SAM for camouflaged-region segmentation and then pass the mask as a soft spatial prior to open-vocabulary classification [31]. The latter setting is relevant to subtle boundaries, but it differs from the present study in both supervision and scope: it targets arbitrary or unseen categories with a language encoder and SAM, whereas WS-DA-DETR learns four predefined apple classes from folder labels and uses no text prompt. We therefore treat VLM-guided localization as a related extension for unseen defect categories rather than as a directly comparable numerical baseline.

## 3. Materials and Methods

### 3.1. RGB Sensing Task and Dataset Protocols

The training labels and inspection outputs differ in spatial detail. Each image provides an apple category and acquisition domain but no human-drawn lesion region. Image-level decisions therefore use folder labels directly, whereas spatial outputs receive only weak, appearance-derived guidance. The notation below keeps those sources distinct.

Let the training set be

(1) $$D={\left\{\left(I_{i},y_{i}^{c},y_{i}^{h},d_{i}\right)\right\}}_{i=1}^{N},$$

where $I_{i}\in R^{3\times H\times W}$ is an RGB image, $y_{i}^{c}\in \left\{0,1,2,3\right\}$ denotes fresh, bruise, rot, or scab, $y_{i}^{h}=0$ denotes healthy, $y_{i}^{h}=1$ denotes defective, and $d_{i}\in \left\{\mathrm{real},\mathrm{synthetic}\right\}$ is the acquisition domain. The defective state is the positive class for health AUC. No training image has a human-provided lesion box or mask.

Spatial outputs comprise query-level defect logits ${\hat{z}}_{i}^{d}$, boxes ${\hat{b}}_{i}$, and a dense defect map $\hat{M}$. The image-level heads produce four-class logits ${\hat{z}}^{c}$, binary health logits ${\hat{z}}^{h}$, and a visible surface-condition score $\hat{q}\in \left[0,1\right]$, where larger values indicate a healthier visible surface. Its complement $1-\hat{q}$ indicates visible surface deterioration, not internal quality, taste, or storage life. A tilde marks appearance-derived pseudo regions, such as $\tilde{M}$ and $\tilde{b}$, separating weak training cues from manual ground truth.

**Figure 1:**
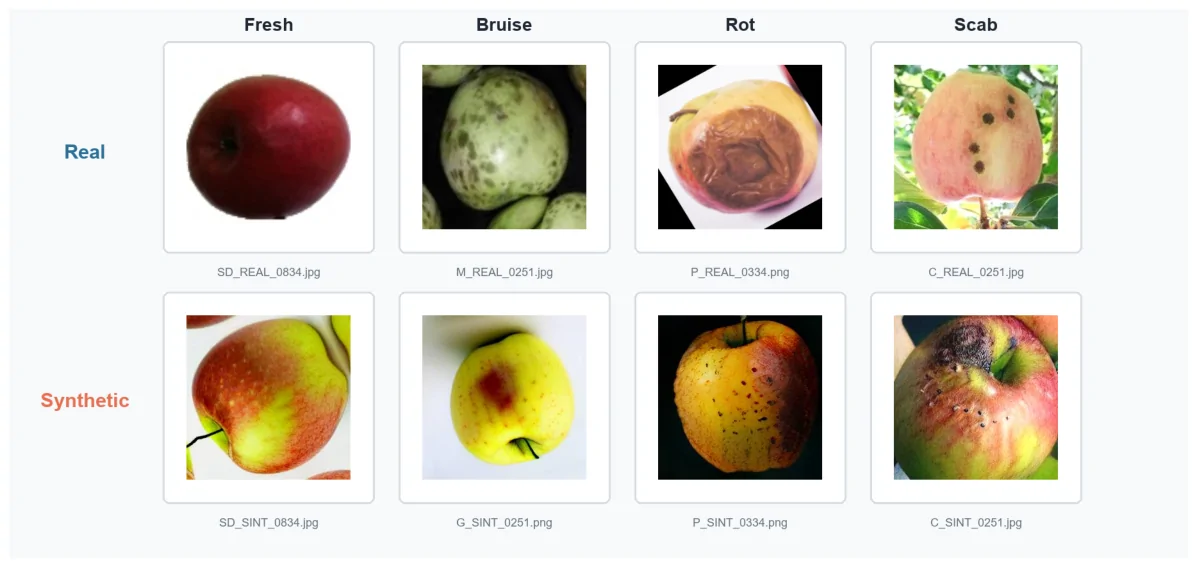
Representative real and synthetic apple images from the local archive. Samples were selected deterministically at one-third of each lexicographically sorted domain/class list. The visible appearance gap motivates separate mixed-domain and cross-domain evaluation.

**Figure 2:**
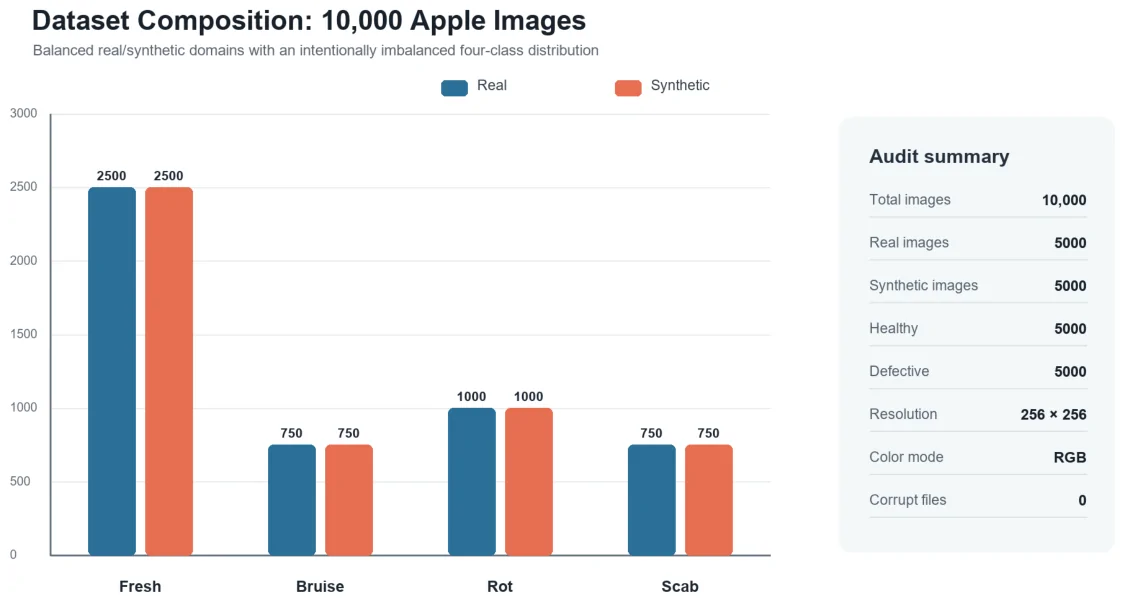
Dataset composition obtained by scanning the local archive. Real and synthetic domains are balanced, while the four-class task is imbalanced toward fresh apples.

The local archive is the apple subset of Healthy-Defective-Fruits, publicly distributed on Kaggle as *Healthy and Defective Fruits* (version 1) [32]. Only apple images are used; the mango branch is excluded. An integrity scan found 10,000 readable RGB images, all at 256×256 pixels. Each domain contains 2500 fresh, 750 bruise, 1000 rot, and 750 scab images (Table 1). The folder name rot_deffect in the synthetic branch is normalized to rot. Figure 1 shows representative images and Figure 2 summarizes the audited counts.

SHA-1 auditing identified 9683 unique file contents and 269 equal-content groups. Exact duplicates are assigned to one split within a domain/class stratum. The primary protocol uses approximately 70%/15%/15% stratified training, validation, and test partitions, yielding 7000/1498/1502 images because equal-content groups and half-count strata are not broken. Two domain-transfer protocols are also used: synthetic-to-real (train/validate on synthetic and test all real images) and real-to-synthetic (the reverse). The split-generation seed is 42. Seeds 42, 3407, and 2026 denote stochastic model-training runs, including initialization, minibatch order, and augmentation, on the frozen split. The same 200 manually evaluated images are used for all three runs. SHA-1 grouping addresses byte-identical files only; visually near-duplicate renderings and repeated views of the same fruit are considered separately in Section 5.3.

A global audit of the 10,000 SHA-1 records, without restricting comparisons to a domain or class, found no equal-content group crossing domains or class labels. The mixed-domain and both transfer manifests also each contain zero equal-content groups spanning train, validation, and test. This audit establishes exact-content separation only. The near-duplicate and cluster-split evaluation is described below.

The global near-duplicate audit used 64-bit pHash distance ≤8 and SSIM ≥0.92. Review of 53 candidate pairs identified 17 groups containing 41 images. Four groups (9 images) crossed class labels and six groups (14 images) crossed domains; these sets can overlap. Review of the four cross-class groups found two label-inconsistent groups, one consistent group, and one unresolved group. Table 2 reports their composition and the repeated cluster-split experiment. Fruit identity remains unavailable for views not recognized as near duplicates.

Across seeds 42, 3407, and 2026, cluster-split ${\mathrm{AP}}_{50}$ is 52.8±1.2%, compared with 54.6±1.2% under the original split. Preprocessing, weak-target rules, training budget, and checkpoint selection are unchanged. Moving 15 near-duplicate images into training reduces the full test cohort from 1502 to 1487 images, while the manually annotated AP evaluation cohort remains fixed at 200 images and 318 lesions. The mean decrease is 1.8 percentage points; all three differences equal 1.8 at the reported precision. This comparison measures sensitivity to the audited split change, not generalization to independent acquisition conditions.

#### Public Data and Evaluation Records

Public source images and folder labels are available from the Kaggle dataset [32]. The study-specific COCO annotations, paired ratings, predictions, and execution records have been recovered and are retained by the authors for upload through the journal submission system. Table 3 distinguishes these records from the public image archive. The COCO image identifiers and frozen manifests use SHA-1 to maintain image identity across annotations and runs.

### 3.2. Manual Evaluation Subset and Annotation Reliability

Spatial localization and visible surface-condition scoring are evaluated on 200 defective images drawn exclusively from the held-out mixed-domain test partition. Table 4 reports the domain-by-class composition. The subset intentionally contains more real than synthetic images while approximately preserving the three defect-class proportions. No threshold or model hyperparameter is selected using these manual test annotations.

Each spatially separated bruise, rot, or scab region is annotated as an individual COCO instance with a bounding box and polygon or uncompressed-RLE mask. Polygons are drawn in LabelMe (v5.2) and converted to the COCO representation used for evaluation. Stems, calyces, specular highlights, and background objects are excluded. Both raters independently assign every image one of five ordered surface-condition levels (0, 0.25, 0.50, 0.75, or 1.0), where larger values indicate a healthier visible surface, and the mean of the two ratings is used only for held-out evaluation. A stratified subset of 40 images (20% of the 200) is independently re-annotated spatially by the second rater before adjudication.

The two raters were postgraduate researchers with prior experience in fruit surface inspection. Annotation was performed in LabelMe (v5.2) under fixed display settings; raters were blinded to model predictions and to each other’s scores. Discrepant boxes/masks (IoU <0.5 or Dice <0.6) were adjudicated by joint review.

Agreement on the five ordered ratings is summarized by quadratic-weighted Cohen’s κ, and numerical-score reliability is summarized by a two-way random-effects, absolute-agreement intraclass correlation coefficient, ICC(2,1), each with a 95% confidence interval. The double-annotated spatial subset is summarized by paired box IoU and mask Dice before adjudication. These statistics describe label reliability and are distinct from agreement between the model and the mean human score.

The first rater annotated all 200 images spatially; the second independently annotated the stratified 40-image spatial subset, and both rated surface condition on all 200 images. The manual labels are excluded from training, threshold selection, loss-function or coefficient selection, pseudo-mask rule design, architecture selection, and checkpoint selection. The lesion-instance counts, selection procedure, and rating anchors are described below.

#### 3.2.1. Additional Manual Evaluation Records

Table 5 reports image counts and lesion-instance statistics. Each disjoint annotated region contributes one instance, whereas mask IoU uses the union of regions within an image.

The subset was sampled deterministically from defective images in the mixed-domain test partition, stratified by domain and class, with sampling seed 42. The 200 SHA-1 identifiers were frozen with the run manifests.

The five surface-condition anchors are as follows: 0.00, severe visible damage affecting more than 60% of the visible surface or deep rot; 0.25, extensive visible lesions affecting approximately 30–60%; 0.50, moderate damage affecting approximately 10–30%; 0.75, slight or localized damage affecting less than 10%; and 1.00, no visually apparent defect. These are ordinal visual-inspection anchors; the area bands are approximate, and deep visible rot also informs the lowest category. Both raters used this scale independently.

#### 3.2.2. Spatial Agreement and Unmatched Lesions

The recovered matching procedure uses greedy one-to-one assignment by maximum box IoU within the same class. At each step, the unmatched pair with the highest box IoU is selected if IoU ≥0.5, and both lesions are removed from further matching. The same matched pairs are used for mask Dice. The previous calculation ignored unmatched lesions, giving box IoU 0.68 and mask Dice 0.72; these are matched-pair-only summaries.

The reanalysis retains this greedy matching procedure and includes unmatched lesions. For image *i*, let $m_{i}$ be the number of matches and let $u_{i}^{\left(1\right)}$ and $u_{i}^{\left(2\right)}$ be the unmatched counts for the two raters. The image-level score is

(2) $$S_{i}=\frac{\sum_{j=1}^{m_{i}}s_{ij}}{m_{i}+u_{i}^{\left(1\right)}+u_{i}^{\left(2\right)}},\;s_{ij}\in \left\{\mathrm{box}\;\mathrm{IoU},\mathrm{mask}\;\mathrm{Dice}\right\}.$$

Each unmatched lesion contributes zero. Images empty for both raters are excluded from lesion agreement and counted separately; an image empty for only one rater scores zero. All 40 double-annotated images are evaluable, with no both-empty image. Mean scores and 95% percentile intervals use image-level resampling of these 40 images, with 10,000 replicates and random seed 42. Table 6 reports the recovered counts and the unmatched-aware results.

### 3.3. Architecture Overview and Surface Defect Token Encoding

**Figure 3:**
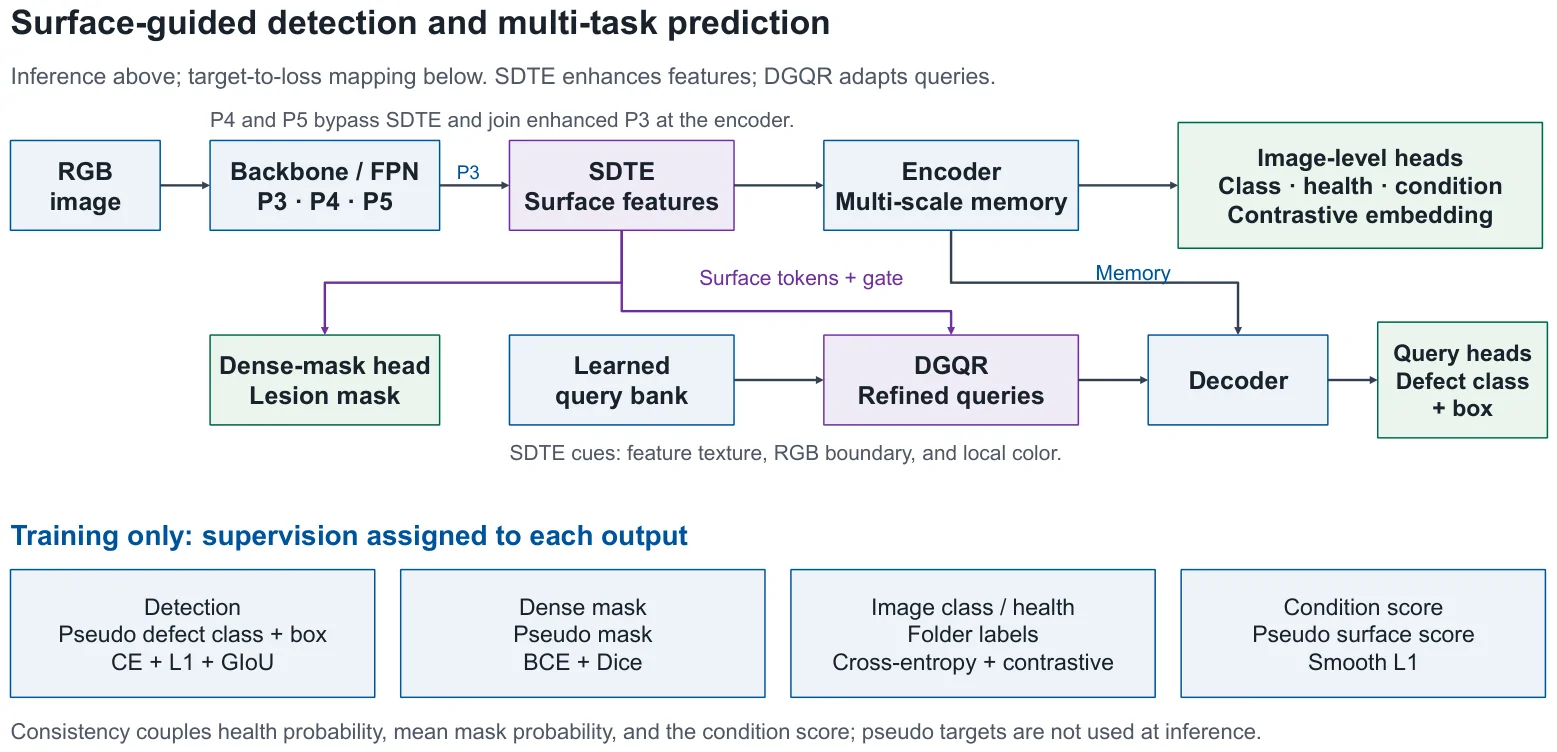
Architecture and supervision of WS-DA-DETR. Solid arrows trace inference: backbone/FPN features enter the encoder, SDTE enhances $P_{3}$, and DGQR conditions learned queries on pooled surface tokens and their gate before decoding. $P_{4}$ and $P_{5}$ bypass SDTE. The decoder supplies class/box predictions, ${\tilde{P}}_{3}$ supplies the dense mask, and mean encoder memory supplies image class, health, condition, and embedding heads. The lower strip maps outputs to training targets and losses; it is not an inference stage. RGB appearance cues are computed separately for SDTE and the fixed pseudo-target generator. Equations and settings are defined in the text.

Figure 3 separates three paths. The feature path extracts multi-scale context, with SDTE enhancing fine surface evidence on $P_{3}$. The query path uses DGQR to adapt learned queries before decoding. Output paths produce query boxes, a dense mask, and image-level decisions from their respective features. Training targets and losses are shown separately below inference.

As summarized in Figure 3, an ImageNet-pretrained ConvNeXt-Tiny backbone extracts feature maps $\left(C_{2},C_{3},C_{4}\right)$ [19]. A feature pyramid [33] maps them to a common dimension D=256:

(3) $$P_{5}=\phi_{5}\left(C_{4}\right),\;P_{4}=\phi_{4}\left(C_{3}\right)+U\left(P_{5}\right),\;P_{3}=\phi_{3}\left(C_{2}\right)+U\left(P_{4}\right),$$

where $\phi_{s}$ is a lateral projection plus smoothing convolution and U is bilinear upsampling. SDTE modifies the highest-resolution $P_{3}$. The resulting surface map, $P_{4}$, and $P_{5}$ are flattened with two-dimensional sine positional encodings and learned level embeddings. To bound attention cost, $P_{3}$ is average-pooled by two before tokenization.

#### Surface Cues and Fusion

SDTE combines a feature-space texture residual, RGB-derived boundaries, and local RGB deviation. None is lesion-specific; the learned gate controls how much this evidence modifies $P_{3}$. The texture cue is

(4) $$T_{f}=\left|{\mathrm{DWConv}}_{3\times 3}\left(P_{3}\right)-P_{3}\right|.$$

Let *Y* denote grayscale intensity. The boundary cue uses fixed Sobel kernels $S_{x}$ and $S_{y}$:

(5) $$B=\sqrt{{\left(S_{x}*Y\right)}^{2}+{\left(S_{y}*Y\right)}^{2}+\epsilon }.$$

The local color-abnormality cue is

(6) $$C={∥I-{\mathrm{AvgPool}}_{21\times 21}\left(I\right)∥}_{2}.$$

For SDTE, *B* and *C* are bilinearly resized to $P_{3}$ resolution and each is divided by its per-image spatial maximum plus $10^{-6}$. This maximum scaling differs from the foreground-percentile scaling of the fixed pseudo-target generator. A learned residual *U* and spatial gate *G* are then computed as

(7) $$\begin{array}{cc} U & =\phi \left([P_{3},T_{f},B,C]\right), \end{array}$$

(8) $$\begin{array}{cc} G & =\sigma \left(\psi (\left[P_{3},B,C\right])\right), \end{array}$$

(9) $$\begin{array}{cc} {\tilde{P}}_{3} & =P_{3}+G⊙U, \end{array}$$

where [·] is channel concatenation, ϕ contains point-wise and 3×3 convolutions, and ψ produces a one-channel gate. The residual connection preserves $P_{3}$, and the gate gives greater weight to locations with local surface deviations.

### 3.4. Multi-Scale Encoding and Defect-Guided Query Refinement

The surface-enhanced representation remains within the feature pyramid. Local deviations are retained in ${\tilde{P}}_{3}$, alongside the larger receptive fields of $P_{4}$ and $P_{5}$. Flattened tokens from the three levels are concatenated and processed by $L_{e}=3$ pre-normalized transformer encoder layers:

(10) $$Z=\mathrm{Encoder}\left(\left[\mathrm{Tok}\left({\tilde{P}}_{3}\right);\mathrm{Tok}\left(P_{4}\right);\mathrm{Tok}\left(P_{5}\right)\right]\right).$$

Standard DETR gives every image the same learned query bank although defect position and extent vary. DGQR uses the preceding surface representation to adapt that bank before decoding. Let $Q\in R^{N_{q}\times D}$ be $N_{q}=20$ learned queries, $T_{s}$ the pooled surface tokens, and $g_{s}$ the flattened SDTE gate. Cross-attention yields

(11) $$\begin{array}{cc} R & =\mathrm{MHA}(Q,g_{s}⊙T_{s},T_{s}), \end{array}$$

(12) $$\begin{array}{cc} \alpha & =\sigma \left(\mathrm{MLP}([Q,R])\right), \end{array}$$

(13) $$\begin{array}{cc} \hat{Q} & =\mathrm{LN}(Q+\alpha ⊙R). \end{array}$$

The decoder combines refined queries with memory *Z*. Query embeddings predict three defect classes plus no-object and normalized $\left(c_{x},c_{y},w,h\right)$ boxes. The dense mask is predicted separately from ${\tilde{P}}_{3}$. The encoder and decoder each have three pre-normalized layers, eight attention heads, feed-forward dimension 1024, and dropout 0.1.

### 3.5. Multi-Task Prediction and Pseudo-Localization Curriculum

#### 3.5.1. Image-Level Outputs

The image heads summarize the complete apple. Global context $\bar{z}={\left|Z\right|}^{-1}\sum_{j}Z_{j}$ feeds the four-class, binary health, and visible surface-condition heads:

(14) $$\begin{array}{cc} {\hat{z}}^{c} & =W_{c}\bar{z}+b_{c},\;{\hat{p}}^{c}=\mathrm{softmax}\left({\hat{z}}^{c}\right), \end{array}$$

(15) $$\begin{array}{cc} {\hat{z}}^{h} & =W_{h}\bar{z}+b_{h},\;{\hat{p}}^{h}=\mathrm{softmax}\left({\hat{z}}^{h}\right), \end{array}$$

(16) $$\begin{array}{cc} \hat{q} & =\sigma (W_{q}\bar{z}+b_{q}). \end{array}$$

Here, $\hat{z}$ denotes pre-softmax logits and $\hat{p}$ denotes class probabilities. Query probabilities are likewise $\mathrm{softmax}({\hat{z}}^{d})$; losses operate on logits, whereas ROC curves and matching class costs use probabilities. The dense mask $\hat{M}$ denotes mask logits and $\sigma (\hat{M})$ denotes pixel probabilities. An MLP projects $\bar{z}$ to a normalized embedding *e*. Supervised contrastive learning [34] pulls samples with the same binary health state together:

(17) $$L_{\mathrm{supcon}}=\sum_{i}\frac{-1}{\left|P(i)\right|}\sum_{p\in P(i)}\log\frac{\exp(e_{i}^{⊤}e_{p}/\tau )}{\sum_{a\neq i}\exp\left(e_{i}^{⊤}e_{a}/\tau \right)}.$$

The binary grouping is deliberately used for contrastive structure while the four-class cross-entropy retains defect-type discrimination. This term is an auxiliary constraint on the shared representation, not a replacement for either classification target.

#### 3.5.2. Fixed Weak Training Targets

Folder labels train image-level classification; decoder, mask, and condition outputs also require appearance-derived targets. With no human lesion annotation for training, conservative appearance responses are computed after augmentation. They are treated as incomplete cues rather than localization ground truth. The deterministic pseudo-target generator receives the augmented RGB image independently of SDTE features, learned weights, manual masks, or model predictions. The generator and SDTE use related appearance cues in separate computational paths. A foreground mask $M_{f}$ is estimated by robust color distance from the median border color. Within $M_{f}$, normalized gradient, local-intensity residual, and local-color residual maps are combined:

(18) $$A=0.35\bar{B}+0.35\bar{T}+0.30\bar{C}.$$

The pseudo generator receives RGB channels on [0,1], before ImageNet standardization. Its luminance is Y=0.2989R+0.5870G+0.1140B, and the local residual is $T=|Y-{\mathrm{AvgPool}}_{17\times 17}\left(Y\right)|$. The local color residual is the Euclidean RGB distance $C=∥I-{\mathrm{AvgPool}}_{21\times 21}{\left(I\right)∥}_{2}$, whose unnormalized scale lies in $\left[0,\sqrt{3}\right]$. Average pooling uses stride one, zero padding of 8 or 10 pixels, respectively, and the full kernel denominator. For an appearance map *V*, foreground scaling is $\bar{V}=\mathrm{clip}\left(\left(V-Q_{0.05}\right)/\left(Q_{0.95}-Q_{0.05}+10^{-6}\right),0,1\right)$; fewer than 16 foreground pixels produce a zero normalized map.

Border pixels provide a median RGB background estimate. Pixels whose color distance exceeds the larger of 0.08 and the 35th percentile are closed with a 7×7 kernel to form $M_{f}$; if the resulting foreground occupies less than 8% of the image, the complete image is used as a conservative fallback. Each appearance map in *A* is robustly scaled between its 5th and 95th foreground percentiles. For a defective image, pixels at or above the 0.82 quantile of *A* within $M_{f}$ are closed with a 5×5 kernel to form $\tilde{M}$; for a fresh image, $\tilde{M}=0$. If thresholding produces no defective pixel, a 5×5 patch around the largest response is used. The smallest rectangle enclosing all retained pixels is $\tilde{b}$. Consequently, multiple distant pseudo regions yield one union-enclosing training box rather than separate instance boxes. Manual evaluation retains one box per actual lesion. For fresh images, the dense-mask target is all zero, every query is no-object, no box regression target is active, and $\tilde{q}=1$. The weak surface-condition target is

(19) $$\tilde{q}=\left\{\begin{array}{cc} 1, & y^{h}=0,\\ \mathrm{clip}\left(1-1.5|\tilde{M}\left|/\right|M_{f}|,0.05,0.95\right), & y^{h}=1. \end{array}\right.$$

Pseudo-target parameters are selected on the frozen validation split, without manual lesion annotations or human condition ratings. The selected quantile is 0.82, cue weights are (0.35,0.35,0.30), and score slope is 1.5; clipping bounds remain 0.05/0.95. Table 7 specifies the discrete grid and selection rule. Validation health macro-F1, computed from folder-derived health labels, is maximized subject to the stability and foreground-coverage constraints. Foreground coverage measures $M_{f}$, before anomaly thresholding, and is therefore a separate foreground-quality check rather than a lesion-area target.

The pseudo regions may cover only part of a lesion. They are used only for training; all headline localization metrics are computed from manual annotations. The training score $\tilde{q}$ is derived from pseudo-mask area. Human ratings are reserved for held-out evaluation. The intrinsic quality of *A*, $\tilde{M}$, and $\tilde{b}$ is evaluated alongside learned-model performance.

### 3.6. Matching, Training Objective, and Implementation Details

Each defective training image supplies at most one weak box. Matching selects the query that balances the weak class cue and geometric agreement with that box, rather than using unavailable human annotations. The selected query minimizes

(20) $$j^{*}=\arg\min_{j}\left[-p_{j}\left(y^{d}\right)+5{∥{\hat{b}}_{j}-\tilde{b}∥}_{1}+2\left(1-\mathrm{GIoU}({\hat{b}}_{j},\tilde{b})\right)\right],$$

where GIoU is the generalized intersection-over-union [35]. Other queries are assigned no-object. Losses follow the output paths in Figure 3: folder labels supervise image class and health; pseudo targets supervise detection, masks, and condition; and contrastive and consistency terms couple the outputs. The objective is

(21) $$\begin{array}{cc} L= & \lambda_{c}L_{\mathrm{class}}+\lambda_{h}L_{\mathrm{health}}+\lambda_{d}L_{\det}+\lambda_{1}L_{\mathrm{box}}+\lambda_{g}L_{\mathrm{GIoU}}\\ \; & +\lambda_{b}L_{\mathrm{mask}\text{-}\mathrm{BCE}}+\lambda_{m}L_{\mathrm{mask}\text{-}\mathrm{Dice}}+\lambda_{q}L_{\mathrm{quality}}+\lambda_{s}L_{\mathrm{supcon}}+\lambda_{sc}L_{\mathrm{consistency}}. \end{array}$$

The consistency loss penalizes disagreement between global and spatial predictions:

(22) $$L_{\mathrm{consistency}}=\mathrm{SL1}\left({\hat{p}}_{\mathrm{def}}^{h},1-\hat{q}\right)+\mathrm{SL1}\left(\mathrm{mean}\left(\sigma \left(\hat{M}\right)\right),1-\hat{q}\right).$$

The first term aligns defect probability with the complement of the surface-condition score. The second uses mean mask probability over the complete image as an activation proxy; it is not lesion fraction unless the fruit fills the image. Classification, detection, box, mask, and condition terms provide task supervision, while contrastive and consistency terms regularize related outputs. Localization-loss weights increase linearly during the first five epochs, delaying full pseudo-label supervision.

#### Training Settings

These settings apply to WS-DA-DETR and its internal ablations; external comparator settings are listed separately in Section 3.7.

Images are resized to 384×384. Training augmentation applies horizontal flipping with probability 0.5, rotation sampled uniformly within $\pm 12^{{}^{\circ}}$, and color jitter (brightness 0.15, contrast 0.15, saturation 0.12, and hue 0.02) with probability 0.8. The model uses dimension 256, 20 queries, a 128-dimensional contrastive projection, three encoder layers, and three decoder layers. The model is trained for 80 epochs with AdamW [36]; the task-head learning rate is $2\times 10^{-4}$, the backbone rate is $2\times 10^{-5}$, weight decay is $10^{-4}$, and the schedule is cosine annealing. Mixed-precision training, a batch size of 12, and gradient clipping at 0.1 are used. The loss weights for image classification, health, detection class, box $L_{1}$, GIoU, mask BCE, mask Dice, surface condition, contrastive structure, and consistency are 1, 1, 1, 5, 2, 1, 1, 0.5, 0.1, and 0.25, respectively. The best checkpoint is selected by validation macro-F1 for binary health classification. All experiments used an NVIDIA RTX 3090 GPU, an Intel Core i9-10900K CPU (Intel Corporation, Santa Clara, CA, USA), 64 GB RAM, PyTorch 1.13, and CUDA 11.7; training took approximately 8 h.

### 3.7. Baseline Adaptation and Fairness Controls

The image-level recognition comparison is presented in the Results. Task-aligned localization comparisons use map-based methods and query-based detectors. For CAM, SEAM, LCTR, CATR, and WeCLIP, a normalized localization map is converted to a mask and then to connected-component boxes using a threshold selected on the validation split only. For DETR-family models, human boxes and masks are never used for training; each model receives the same fixed RGB-derived pseudo box and mask as WS-DA-DETR. The mask and surface-condition outputs use the dense-mask and score heads specified for each model family in Table 8, with the same weak targets.

The unified protocol fixes the mixed-domain split, the manual test subset, 384×384 inputs, augmentation, optimizer family, 80-epoch budget, seeds 42/3407/2026, and validation health macro-F1 checkpoint selection. The exact backbone/FPN, pretrained weight identifiers, mask and score head inputs, loss coefficients, and learning rates for each external detector are described in Section 3.7 and Table 9 and Table 10. Localization-map thresholds and morphological parameters are frozen from training or validation data and are not tuned on the 200-image manual test subset.

The internal ablations keep the implemented mask/score heads and training scaffold fixed. The four external weak DETR-family comparators were rerun under a shared task protocol, with architecture and pretraining differences listed in Table 9. Their results compare adapted systems rather than isolate the effect of the backbone. WFS-DETR uses large-scale pretraining and object-integrity-aware multiple-instance learning for weakly supervised few-shot detection [29]. Omni-DETR uses teacher–student training with matching-based pseudo-label filtering across mixed annotation types [30]. These two methods were not included in the numerical comparison because their additional pretraining or annotation requirements were not matched to the folder-only apple task.

#### 3.7.1. Head Definitions and Matched Comparator Records

For WS-DA-DETR and the internal ablations, the dense-mask head is a 3×3 convolution (256→128), GroupNorm with 16 groups, GELU, and a 1×1 convolution (128→1). It operates on ${\tilde{P}}_{3}$ for the full model and $P_{3}$ when SDTE is absent. The output is a logit map. For manual evaluation, sigmoid probabilities are thresholded at 0.5 at the native output resolution; the binary mask is then resized to the manual annotation dimensions by nearest-neighbor interpolation. The condition head is a linear projection (256→1) of mean encoder memory followed by sigmoid. The ImageNet initialization is torchvision ConvNeXt-Tiny DEFAULT (IMAGENET1K_V1); the newly added FPN, transformer, and prediction heads are initialized for training.

All four evaluated external detectors use the same dense-mask head on $P_{3}$ and a linear condition-score head as the proposed model, with the common task-loss coefficients specified in Section 3.6. The task-head and backbone learning rates are $2\times 10^{-4}$ and $2\times 10^{-5}$, respectively. Table 10 summarizes the recorded repositories and implementation settings. Weak DETR uses the facebookresearch/detr weight provider at commit 4e1a3f9, initialized from detr-r50-e632da11.pth. The evaluated Weak Deformable DETR + DN adaptation uses DN-DETR denoising with 100 groups, noise scale 0.4, and loss coefficient 1.0. Weak RT-DETR uses ResNet-50 with configuration rtdetr_r50vd_6x_coco.yml.

Weak DETR, Weak Deformable DETR + DN, Weak DINO, and Weak RT-DETR completed unified reruns for seeds 42, 3407, and 2026. Section 4.3 reports those results. The reruns share the image split, 200-image manual subset, augmentation, frozen weak targets, optimizer, task learning rates, 80-epoch budget, and validation health macro-F1 checkpoint criterion. Backbone and pretrained-weight differences remain as documented in Table 9. WFS-DETR and Omni-DETR are discussed as related methods rather than assigned unmatched apple-domain results.

Weak Deformable DETR + DN denotes deformable attention with added DN-DETR-style training denoising: 100 groups, noise scale 0.4, and coefficient 1.0. The denoising-disabled control sets the group count to zero while retaining the architecture, weak targets, and training budget. Its ${\mathrm{AP}}_{50}$ is 43.9±1.7%, compared with 45.3±1.6% with denoising, a mean gain of 1.4 percentage points (Section 4.3). Backbone and pretrained-weight differences remain, so the external comparison evaluates adapted systems rather than architecture-only effects.

The external condition heads are evaluated against the same two-rater mean scores on the same 200 images and three training seeds as WS-DA-DETR. The external condition-score comparison reports the Pearson correlation, Spearman correlation, MAE, and RMSE under the common aggregation convention. These comparisons complement the deterministic pseudo-score and internal-ablation analyses.

#### 3.7.2. Use of Generative AI-Assisted Tools

OpenAI Codex (GPT-5.6) assisted with initial code scaffolding, LaTeX organization, and language editing. The authors checked all generated text and code and are responsible for the study design, analysis, and reporting.

## 4. Results

### 4.1. Evaluation Protocol and Image-Level Recognition

Image-level recognition is evaluated with accuracy, macro-precision, macro-recall, and macro-F1. Binary health assessment additionally uses receiver operating characteristic area under the curve (AUC), with defective apples as the positive class. These measures describe class decisions but do not establish whether the predicted evidence overlaps a lesion. Spatial performance is therefore evaluated separately on a held-out, manually annotated subset of defective test images under COCO-style bruise, rot, and scab categories [37]. ${\mathrm{AP}}_{50}$ uses a box intersection-over-union (IoU) threshold of 0.50, whereas ${\mathrm{AP}}_{50:95}$ averages thresholds from 0.50 to 0.95 in steps of 0.05. Localization recall@0.5 is the proportion of manual lesion boxes matched one-to-one by a same-class prediction at IoU ≥0.5. Recall uses a score threshold of 0.3 and a maximum of 20 detections per image.

Dense localization and condition scoring use complementary measures. Mask IoU is computed per image after unioning all manual lesion masks and thresholding native-resolution mask probabilities at 0.5, then resizing the binary prediction by nearest-neighbor interpolation; the reported value is the mean over annotated images. The visible surface-condition score is compared with the mean of two independent human ratings using Pearson *r*, tie-aware Spearman ρ, mean absolute error (MAE), and root-mean-square error (RMSE). Percentile bootstrap intervals are computed over images. Rater–rater weighted κ and ICC are reported separately so that label reliability is not conflated with model agreement. Parameter count, multiply–accumulate operations (MACs), latency, and frames per second characterize model complexity and model-only inference cost rather than complete sorting-line throughput.

When human annotations are absent, the evaluator prefixes localization outputs with pseudo_ and marks them as proxy values. These proxy measures are used for debugging and are excluded from the reported spatial-evaluation and ablation results; they are not substitutes for manual spatial evaluation. Aggregate model results are reported as mean ± standard deviation over seeds 42, 3407, and 2026 where applicable. Human-rating and spatial-agreement confidence intervals reported below are image-level bootstrap intervals; the single-valued Spearman, MAE, and RMSE entries refer to pooled held-out predictions. Section 4.3 reports individual-run and subgroup results and paired bootstrap intervals for selected method differences. These intervals concern the evaluated systems and test images, rather than isolating architecture or establishing performance in unseen acquisition settings.

Under the mixed-domain split, Table 11 compares image-level recognition by ResNet, EfficientNet, ConvNeXt, ViT, Swin, and WS-DA-DETR. WS-DA-DETR reaches 89.5% four-class accuracy and 96.3% health AUC, 1.3 and 1.0 percentage points above the corresponding Swin-T values. The macro-F1 difference is smaller: 87.4% for WS-DA-DETR and 86.4% for Swin-T. Task-aligned spatial results are reported in Section 4.3 under the shared protocol in Table 8.

**Figure 4:**
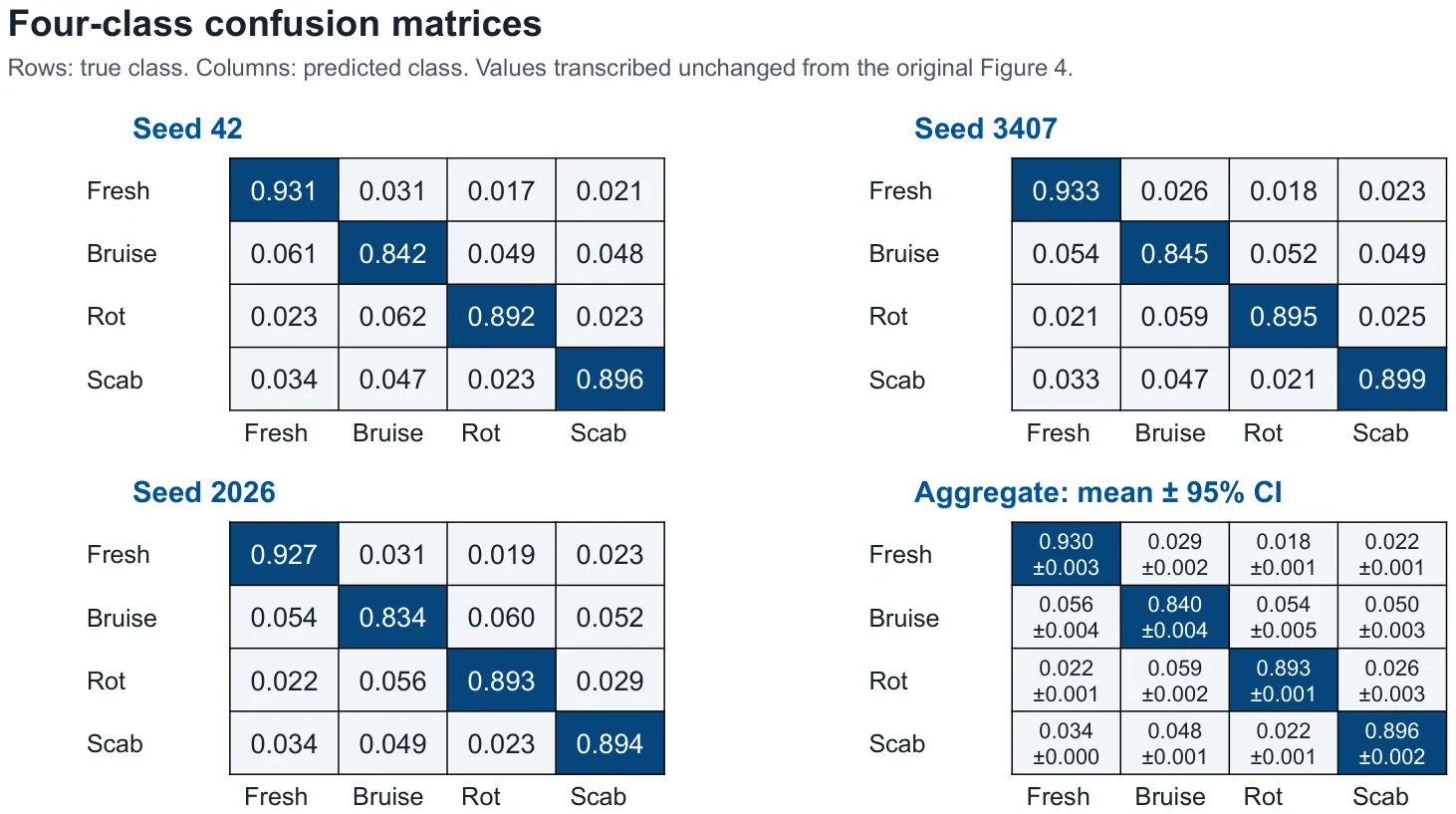
Row-normalized four-class confusion matrices for seeds 42, 3407, and 2026, and the original aggregate summary. Dark diagonal cells emphasize correct classifications and are not a continuous color scale. Class order is fresh (healthy), bruise, rot, and scab.

**Figure 5:**
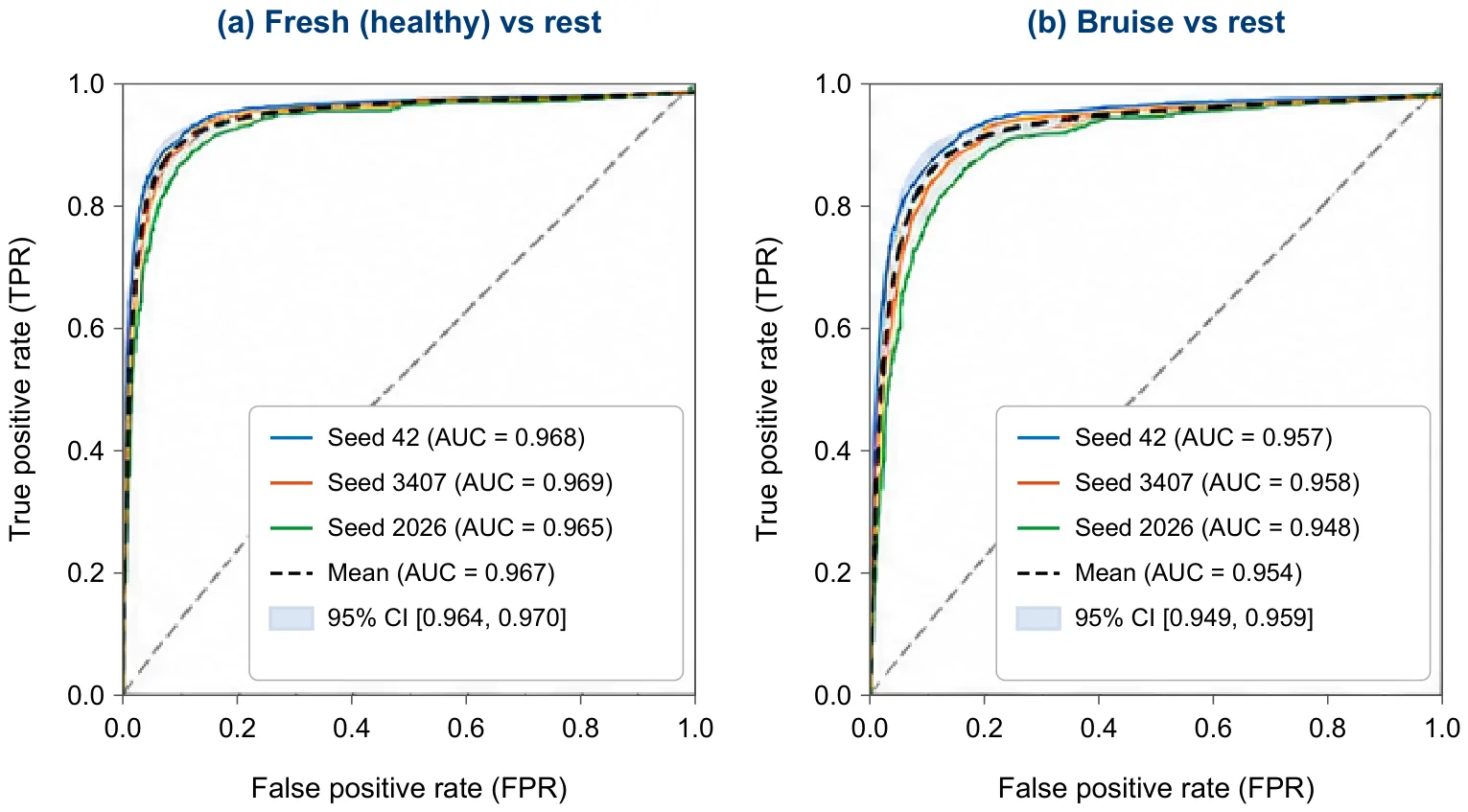

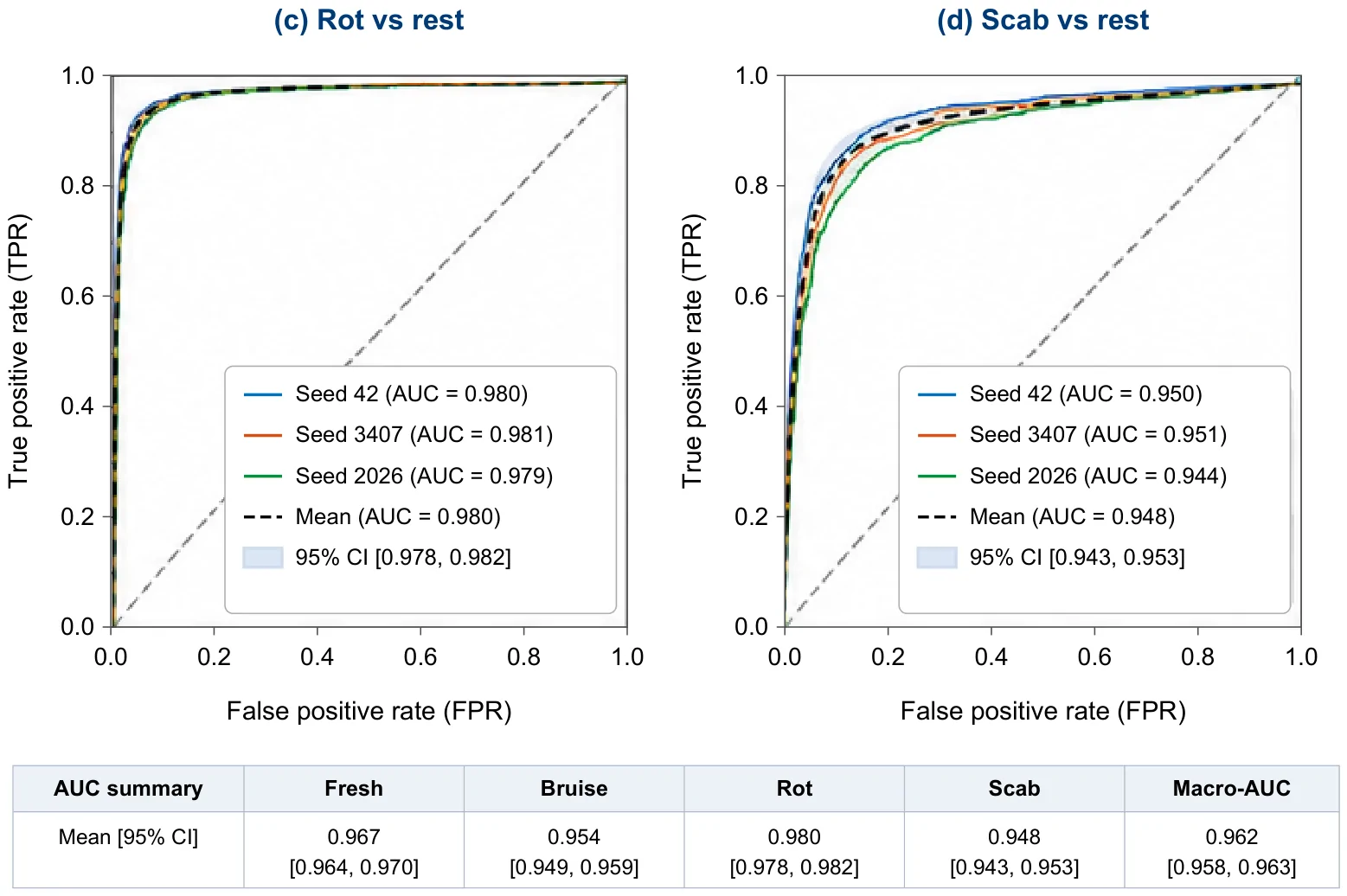
One-versus-rest ROC curves for (**a**) fresh (healthy) and (**b**) bruise. Original one-versus-rest ROC curves for (**c**) rot and (**d**) scab, with an AUC summary across three seeds. Curves and previously reported 95% bootstrap intervals are retained without recalculation. These are four-class, not binary-health AUCs.

Figure 4 and Figure 5 separate the original confusion matrices from one-versus-rest ROC analysis. These four-class diagnostics differ from binary health AUC. The plotted intervals use 1000 test-set bootstrap resamples with seed 42. The curves and their interval values are retained from the original analysis; separating the graphics changes presentation rather than the statistical calculation.

### 4.2. Intrinsic Quality of the Pseudo-Localization Signal

To quantify the spatial information supplied by the fixed pseudo-target generator, the raw anomaly map *A*, its thresholded mask before morphology, and the final post-processed pseudo mask and box are evaluated directly against the held-out manual annotations. Pixel AUPR summarizes the unthresholded anomaly map; mask IoU evaluates binary spatial overlap; and ${\mathrm{AP}}_{50}$ plus recall@0.5 evaluate the enclosing pseudo box. The quantile, foreground rule, and morphology are frozen without access to the manual test annotations. Table 12 summarizes these measurements and the downstream learned-model results.

Pixel AUPR of the continuous raw anomaly map *A* is 0.41; at the frozen threshold, the corresponding mask reaches 28.5% IoU and its enclosing box reaches 31.2% ${\mathrm{AP}}_{50}$. After morphological post-processing, the pseudo mask reaches 35.8% IoU and the enclosing pseudo box reaches 38.4% ${\mathrm{AP}}_{50}$ on the same 200-image subset. These measurements establish the spatial quality of the fixed curriculum. The learned WS-DA-DETR improves mask IoU by 12.9 percentage points and box ${\mathrm{AP}}_{50}$ by 16.2 points over the final pseudo targets.

### 4.3. Defect Localization and Surface-Condition Assessment

Table 13 separates task-aligned spatial evaluation from the image-level context in Table 11. Map-based weak-localization methods obtain boxes and masks from their documented localization maps; query-based methods receive the same RGB-derived pseudo targets and use the output heads described in Table 8. For WS-DA-DETR, box localization reaches 54.6% ${\mathrm{AP}}_{50}$ and 32.3% ${\mathrm{AP}}_{50:95}$, while dense overlap and localization recall are 48.7% and 74.5%, respectively. Surface-condition agreement and rater reliability are reported separately in Table 14.

Quadratic-weighted κ=0.71 and ICC(2,1) =0.74 indicate consistent inter-rater agreement on the five-level scale. Pre-adjudication unmatched-aware mean box IoU and mask Dice on the 40-image double-annotated subset are 0.59 and 0.63, respectively. Model–human Pearson r=0.68 (95% CI [0.61,0.74]) is interpreted in light of the observed label reliability. The reported confidence intervals are image-level bootstrap intervals; the ±0.03 attached to Pearson *r* summarizes variation across the three model seeds. The manual subset contains 318 lesions in 200 images, as detailed in Section 3.2. Against the same mean human ratings, the deterministic pseudo score yields Pearson r=0.49, Spearman ρ=0.47, MAE =0.21, and RMSE =0.26. The model reaches r=0.68, ρ=0.66, MAE =0.14, and RMSE =0.18. The corresponding improvements are 0.19 in each correlation, 0.07 lower MAE, and 0.08 lower RMSE.

Including unmatched lesions reduces mean box agreement from the matched-pair-only value of 0.68 to 0.59 and mask Dice from 0.72 to 0.63. The updated values in Table 6 and Table 14 account for lesions identified by only one rater. Table 15 compares condition-score performance across the adapted query-based systems.

WS-DA-DETR has the highest reported correlations and lowest errors among these adapted systems. Relative to Weak DINO, the strongest external condition-score comparator, mean Pearson *r* is higher by 0.07, Spearman ρ by 0.08, and MAE and RMSE are lower by 0.02 and 0.03, respectively. No paired significance test is reported for these external condition-score differences.

#### 4.3.1. Individual Runs and Subgroup Evaluation

Table 16 lists individual-run spatial and condition-score results. Recall uses same-class one-to-one matches at IoU ≥0.5, a prediction-score threshold of 0.3, and at most 20 detections per image. For seed *s* and subgroup *g*, $R_{s,g}=100\;TP_{s,g}/N_{g}$; the table reports the mean over the three seeds. Mean TP counts across seeds are 55, 89, and 93 for bruise, rot, and scab, and 155 and 82 for real and synthetic images. Each partition therefore sums to a mean of 237 TPs over 318 lesions, giving 100(237/318)=74.5283%, reported as 74.5%. The seed-specific total TP counts are 242, 236, and 233, giving recalls of 76.1%, 74.2%, and 73.3%. The sample SD computed before rounding is 1.4 percentage points. Rounded subgroup percentages need not reproduce the last displayed digit of an aggregate weighted average.

Table 17 provides class- and domain-specific localization results.

Rot gives the highest class-specific ${\mathrm{AP}}_{50}$ and mask IoU, while synthetic images yield higher spatial scores than real images.

#### 4.3.2. Paired Uncertainty and Pseudo-Score Comparison

Paired image bootstrap resamples the same image identifiers for compared methods, retaining the lesions, both ratings, and seed predictions associated with each image. Sampling is stratified by domain and class. Each replicate recomputes AP from resampled detections and annotations, mean image-wise IoU, lesion recall, and rating association, and the seed-specific differences are averaged. Percentile limits at 2.5% and 97.5% define the 95% intervals in Table 18. These intervals quantify image-sampling uncertainty conditional on the fitted runs, separately from between-seed standard deviations. The analysis used 10,000 stratified image-level bootstrap replicates with random seed 42. Repeated images were retained with unique replicate identifiers; failed replicates were discarded and redrawn.

The model improves on Weak DETR by 9.5 ${\mathrm{AP}}_{50:95}$ points (95% CI [7.8,11.2]), 12.2 mask-IoU points ([10.1,14.3]), and 12.0 recall points ([9.6,14.4]). Relative to the final pseudo targets, the gains are 12.9 mask-IoU points ([10.7,15.1]), 16.2 ${\mathrm{AP}}_{50}$ points ([13.9,18.5]), and 0.19 in the condition-score Pearson correlation ([0.12,0.26]). These paired intervals exclude zero for the specified comparisons. The pseudo score is computed on the unaugmented held-out images with the frozen foreground, mask, and score rules. The rating-reliability intervals in Table 14 used 10,000 image-level bootstrap replicates with random seed 42. All 200 paired-rating records were retained in the sampling frame, and no failed-replicate handling rule was applied. For spatial agreement, the sampling frame is the frozen 40-image double-annotated subset. Each of 10,000 bootstrap replicates, with random seed 42, draws 40 image identifiers with replacement. The pre-adjudication image-level box-IoU and mask-Dice scores include zero contributions for unmatched lesions, as defined in Section 3.2. The replicate statistic averages the 40 drawn image-level values, and the 2.5% and 97.5% percentiles define the reported interval. This image-based spatial bootstrap is separate from the 200-image rating bootstrap.

**Figure 6:**
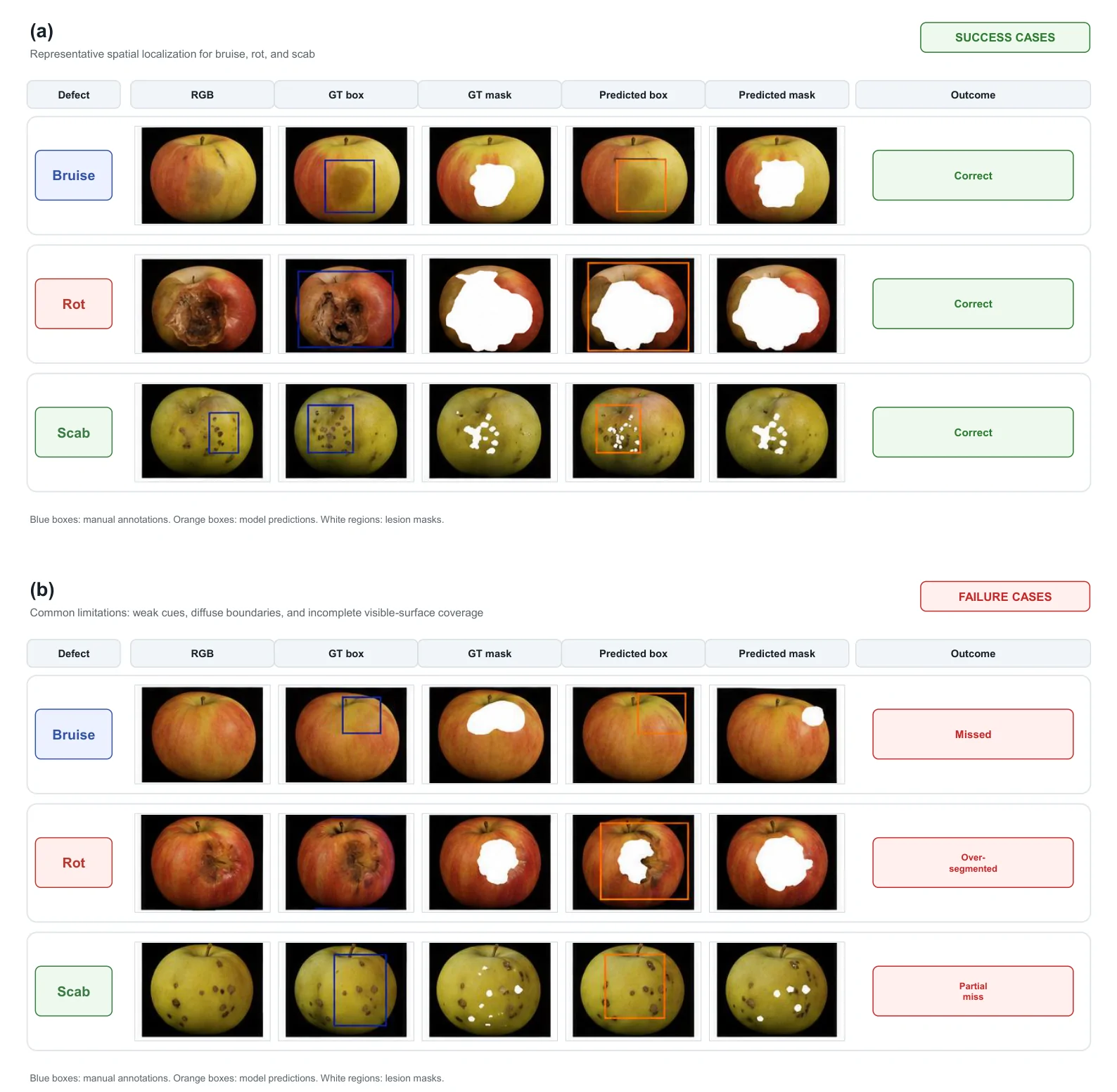
Qualitative localization: (**a**) Representative successful cases and (**b**) representative failure cases. Each row shows the RGB input, manual box and mask, predicted box and mask, and the localization outcome. Blue and orange rectangles denote manual and predicted boxes. The selected failures illustrate a missed bruise, over-segmented rot, and partially missed scab. The panels illustrate spatial localization only.

Figure 6 places these aggregate measures beside representative spatial predictions. Panel (a) shows successful spatial localization, and panel (b) shows typical failure modes. A faint bruise is largely missed, a rot region is over-segmented, and scattered scab lesions are only partly recovered. These panels illustrate localization only; aggregate condition-score evaluation is reported in Table 14.

### 4.4. Ablation Study

Table 19 places the four detector configurations beginning with the internal multi-task weak DETR baseline, followed by SDTE-only, DGQR-only, and SDTE+DGQR, so that the two modules can be compared independently and jointly. These rows keep the image heads, pseudo targets, base multi-task losses, data splits, epoch budget, and checkpoint rule fixed; the later rows then add contrastive and consistency terms without changing the architecture. The internal ablation baseline uses the proposed multi-task training scaffold and is therefore distinct from the task-aligned Weak DETR comparator in Table 13.

### 4.5. Cross-Domain Generalization

The real and synthetic halves are balanced by image count but not by low-level appearance. Table 20 therefore reports synthetic-to-real and real-to-synthetic transfer apart from mixed-domain testing, with no target-domain test image used for model selection. Synthetic-to-real transfer is the harder direction: WS-DA-DETR exceeds ConvNeXt-Tiny by 5.8 percentage points in accuracy and 6.5 points in macro-F1, yet its absolute performance remains below the mixed-domain result. In the reverse direction, the differences are 2.8 and 3.3 points. The asymmetry is consistent with the wider appearance variation observed in the real-image diagnostics, although those descriptors do not establish a single cause for the transfer gap.

### 4.6. Model Inference Efficiency

Relative to the weak DETR baseline, WS-DA-DETR adds 4.2 M parameters and 1.3 G MACs (Table 21). The added computation accompanies the surface encoder, query adaptation, and multiple prediction outputs. Batch-one model timing is 30.2 ms per image, or 33.1 FPS, on the RTX 3090. This value characterizes the neural-network forward path after warm-up and does not include image acquisition, data transfer, fruit tracking, or actuator latency.

## 5. Discussion

### 5.1. Domain Shift and Component Interpretation

**Figure 7:**
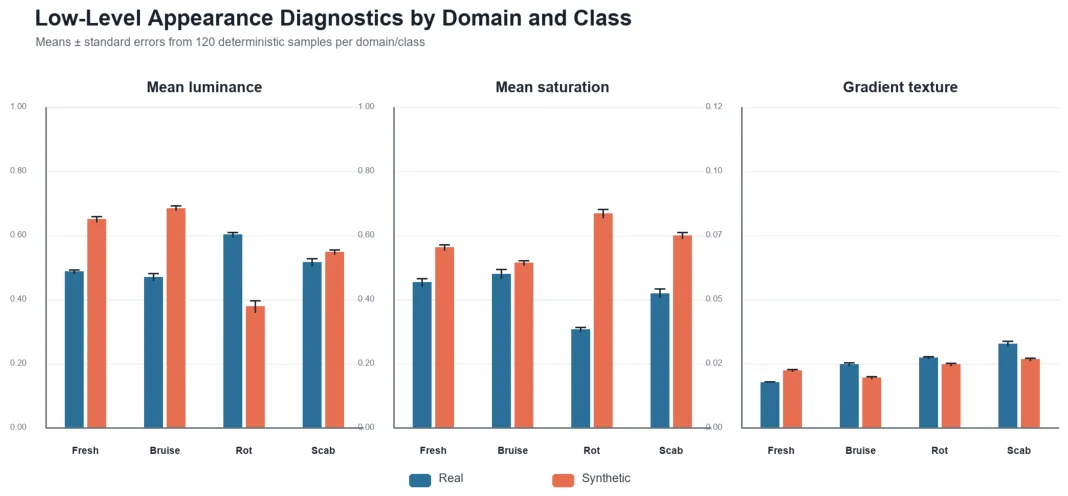
Low-level appearance diagnostics from 120 local samples per domain/class selected with seed 42. Bars show means and error bars show standard errors for luminance, HSV saturation, and mean absolute grayscale gradient. The plots describe class-dependent real/synthetic appearance shifts and motivate cross-domain evaluation.

Figure 7 summarizes three image descriptors computed from 120 samples in every domain/class combination using a fixed pseudorandom seed of 42. Equal cell sizes make the domain/class comparisons balanced while limiting descriptor computation; the plots are descriptive and were not used for model selection or hypothesis testing. The domain gap is also class dependent. Sampled synthetic rot images, for example, have mean saturation of 0.667 compared with 0.306 for real rot, while their mean luminance is 0.378 compared with 0.602. Texture differences are smaller and can reverse direction across classes. A mixed-domain split can therefore reward cues that are correlated with acquisition domain as well as defect type, which is why the transfer protocols in Table 20 are interpreted separately.

The experiments evaluate the pseudo-target generator, the two architectural modules, and the auxiliary losses separately. First, Table 12 measures the fixed anomaly map and pseudo targets before learning; this determines how much spatial signal is already present in the curriculum. Second, the four detector rows beginning with the internal multi-task weak DETR baseline compare neither module, SDTE only, DGQR only, and both modules while holding the remaining training design fixed. Third, the later rows assess auxiliary regularization.

Relative to the internal multi-task weak DETR baseline, the SDTE-only, DGQR-only, and joint configurations increase health macro-F1 by 0.5, 0.4, and 0.8 percentage points; ${\mathrm{AP}}_{50:95}$ by 2.7, 2.0, and 5.5 points; and surface-condition *r* by 0.06, 0.04, and 0.11, respectively. The corresponding mean-scale interaction contrasts are −0.1 health-F1 point, +0.8 ${\mathrm{AP}}_{50:95}$ point, and +0.01 in *r*. These contrasts summarize interactions on the mean-performance scale. Adding the contrastive and consistency losses increases ${\mathrm{AP}}_{50:95}$ from 31.0% to 32.3% and surface-condition correlation from 0.63 to 0.68 and is consistent with the role of cross-output regularization.

### 5.2. Proof of Concept for RGB Surface Inspection

This study is a proof of concept using a public archive of centered, single-apple RGB images. It has not evaluated a physical sorting line, fruit rotation, occlusion, independent cultivars, or changes in camera and illumination. Independent acquisition and fixed-checkpoint external evaluation are specified below. The RGB setting combines simple image acquisition with several levels of output. Each forward pass returns a defect class, a spatial estimate, binary health, and a visible surface-condition score. The box and mask provide a check on the region associated with a rejection decision, while the continuous score can distinguish visible condition within the broader healthy/defective grouping. These outputs do not remove the need for operational quality control, but they offer more inspectable evidence than a folder-trained classifier alone.

Model-only latency is 30.2 ms per image, corresponding to 33.1 FPS on the RTX 3090. This rate describes the network after warm-up, not the throughput of a complete sorting installation. Image exposure, data transfer, fruit tracking, synchronization, and actuation would all contribute additional delay. The tested hardware is also a desktop GPU, so the reported value should not be transferred directly to an edge processor without a new measurement.

Acquisition stability remains important even when the model is trained on both real and synthetic images. The class-dependent luminance and saturation differences suggest that exposure and color calibration can change the cues available to the surface encoder. An RGB system is attractive because it avoids specialized spectral hardware; it does not observe internal damage or guarantee that every faint lesion is separable from normal peel variation. Deployment would therefore require camera geometry and illumination to be controlled and performance to be checked on the cultivars and operating conditions of the target line.

An independent acquisition study has not yet been conducted. The planned validation will collect approximately 300 local Fuji and Red Fuji apple images using an industrial camera and fixed LED ring illumination. The model checkpoint will remain frozen for evaluation against newly obtained manual annotations. These planned images and results are not included in the present experiments.

### 5.3. Limitations

The manual localization subset contains 200 defective images and does not characterize lesion-level false positives on healthy fruit. Although study-specific annotations, ratings, predictions, and execution records have been recovered, independent reproduction requires their release together with the image identifiers and evaluation settings. They are designated for upload through the journal submission system, separately from the public source images.

The data represent centered, single-apple RGB images rather than fruit moving through a packing line or appearing under occlusion. Exact duplicate files were kept within one split. The near-duplicate audit identifies 17 groups and 41 images. The cluster-split experiment lowers mean ${\mathrm{AP}}_{50}$ by 1.8 points across three runs on the unchanged manual evaluation subset. Two cross-class groups are label-inconsistent and one remains unresolved; the review does not establish label correctness for the full archive. Fruit identity is also unavailable, so unrecognized repeated views may remain. The real and synthetic domains broaden appearance relative to either source alone, yet they do not substitute for independent cultivars, cameras, illumination settings, or packing-line data. Generalization to those conditions remains unmeasured.

The weak spatial supervision imposes a second set of limits. Appearance-based pseudo targets can miss faint lesions, respond to stems or highlights, and enclose multiple separated regions within one box. The qualitative examples confirm that incomplete and over-extended predictions persist even when the image-level decision is plausible. In addition, the continuous output measures visible surface condition only; it is not an estimate of internal damage, taste, storage life, or the unseen side of the fruit. Finally, both the accuracy values and model-only latency belong to the evaluated data and hardware. Independent spatial annotations and end-to-end timing would be required before operational use in a different setting.

The present vocabulary is also closed: fresh, bruise, rot, and scab are specified during training, and the model has no language pathway for naming an unseen defect. VLM-guided segmentation can use semantic prompts and large pretrained representations to address novel categories or weak boundaries [24,31]. Extending the current sensor pipeline in that direction would require a separate open-set protocol, unknown-defect test data, prompt design, and computational-cost evaluation; the present results do not establish open-vocabulary performance.

## 6. Conclusions

This study asked whether folder-labeled RGB apple images could provide more than a category decision. Under the evaluated protocols, WS-DA-DETR produced defect recognition, weak box and mask localization, binary health assessment, and a visible surface-condition score without using human spatial annotations for training. Its surface encoder incorporates texture, boundary, and color-deviation evidence into the high-resolution feature map, after which query refinement makes the DETR query bank responsive to the current image. The two stages form a single route from local appearance deviation to set prediction rather than separate add-on modules.

Mixed-domain four-class accuracy was 89.5%, while ${\mathrm{AP}}_{50}$ on the manually annotated subset was 54.6% and the Pearson correlation with human surface-condition ratings was 0.68. Batch-one inference reached 33.1 FPS on the tested GPU. The cross-domain gap and the observed failures on faint, scattered, or over-segmented lesions show that these results remain tied to the available images, acquisition conditions, and hardware. Within this archive-based proof of concept, the results describe class, spatial, and visible-condition outputs learned from folder labels and appearance-derived targets. The 200-image manual subset supplies evaluation evidence. The pseudo-score comparison and paired intervals support improvement beyond the fixed training targets on this evaluated subset. Three-seed cluster-split ${\mathrm{AP}}_{50}$ is 52.8±1.2%, a 1.8-point decrease on the same manual subset. The external score comparison favors WS-DA-DETR on the reported correlation and error measures, while differences in architecture and pretraining limit causal attribution. Independent acquisition and end-to-end validation remain necessary before deployment.

## Figures and Tables

**Table 1:** Composition of the apple dataset by domain and class.

Domain	Fresh	Bruise	Rot	Scab	Total
Real	2500	750	1000	750	5000
Synthetic	2500	750	1000	750	5000
Total	5000	1500	2000	1500	10,000

**Table 2:** Near-duplicate audit and three-seed cluster-split evaluation. ${\mathrm{AP}}_{50}$ and its decrease are in percentage points.

Audit/Experiment Item	Result
Confirmed near-duplicate groups/images	17/41
Groups spanning class labels	4 groups; 9 images
Groups spanning domains (overlap allowed)	6 groups; 14 images
Cross-class review	2 inconsistent; 1 consistent; 1 unresolved
Seeds and runs	42, 3407, 2026; three runs
Full test cohort	1487 images after moving 15 near duplicates into training
Manual AP evaluation cohort	200 images and 318 lesions, unchanged
Original ${\mathrm{AP}}_{50}$ by seed; mean ± SD	55.9, 54.4, 53.5; 54.6±1.2
Cluster-split ${\mathrm{AP}}_{50}$ by seed; mean ± SD	54.1, 52.6, 51.7; 52.8±1.2
Original minus cluster ${\mathrm{AP}}_{50}$	1.8 for each seed at displayed precision; mean 1.8

**Table 3:** Source images and recovered study-specific evaluation records. Author-held records are designated for upload through the journal submission system.

Record	Scope	Availability
Public fruit images	RGB images and folder labels	Kaggle: Healthy and Defective Fruits, version 1
Frozen splits	Mixed-domain and both transfer protocols	Seed-42 project manifests
Manual annotations	200 images; 318 lesions; COCO boxes and masks	Recovered COCO JSON and frozen SHA-1 image identifiers
Independent ratings	Two five-level ratings for all 200 images	Recovered paired-rating CSV
Per-image predictions	Seeds 42, 3407, 2026	Recovered COCO-format detections and score vectors
Execution/model records	Bootstrap outputs, logs, checkpoints	Recovered bootstrap CSV, training logs, and best checkpoints

**Table 4:** Composition of the manually annotated evaluation subset. The total is 200 defective images.

Domain	Bruise Images	Rot Images	Scab Images	Total Images
Real	40	50	40	130
Synthetic	25	30	15	70
Total	65	80	55	200

**Table 5:** Image and lesion counts in the 200-image manual evaluation subset. Each disconnected lesion is one instance.

Domain	Class	Images	Lesion Instances	Lesions/Image: Mean, Median, Range
Real	Bruise	40	48	1.20, 1, 1–3
Real	Rot	50	72	1.44, 1, 1–4
Real	Scab	40	95	2.38, 2, 1–6
Synthetic	Bruise	25	29	1.16, 1, 1–2
Synthetic	Rot	30	41	1.37, 1, 1–3
Synthetic	Scab	15	33	2.20, 2, 1–4
Total	All defects	200	318	1.59, 1, 1–6

**Table 6:** Pre-adjudication spatial agreement including unmatched lesions on the 40-image double-annotated subset.

Record	Estimate/Count	95% CI
Matched pairs	52	–
Unmatched lesions, rater 1/rater 2	7/9	–
Images empty for both/evaluable	0/40	–
Unmatched-aware mean box IoU	0.59	[0.52, 0.66]
Unmatched-aware mean mask Dice	0.63	[0.56, 0.70]

**Table 7:** Validation-only pseudo-target parameter search. The weight constraint defines 15 ordered triples; the full quantile–weight–slope product contains 225 settings.

Parameter/Selection Item	Values and Criterion
Quantile	{0.75,0.80,0.82,0.85,0.88}; selected 0.82
Score slope	{1.2,1.5,1.8}; selected 1.5
Ordered cue weights	$w_{B}+w_{T}+w_{C}=1$, each in {0.20,0.30,0.35,0.40,0.50}: 15 triples; selected (0.35,0.35,0.30)
Stability	Pseudo-mask Jaccard under ±0.05 weight perturbations; validation-batch mean ≥0.85
Foreground coverage	Mean $|M_{f}|/\left(HW\right)$ over validation defective images; admissible [0.25,0.75]; observed 0.48
Selection and tie-breaking	Maximize validation health macro-F1 subject to stability and coverage; ties use lowest mean pseudo-mask area variance
Search and sensitivity records	Validation IDs frozen with seed 42; candidate scores logged. Perturbing weights by ±0.05 and quantile by ±0.02 changes ${\mathrm{AP}}_{50}$ by at most 1.1 points

**Table 8:** Training supervision, spatial outputs, and evaluation scope of the comparison models.

Model Family	Training Supervision	Spatial and Score Outputs	Evaluation
Classification backbones	Folder classes and binary health labels	Image class and health	Image-level recognition
CAM, SEAM, LCTR, CATR, WeCLIP	Image labels; validation-only map thresholding	Thresholded masks and connected-component boxes	Unified three-seed localization
Weak DETR, Deformable DETR + DN, DINO, RT-DETR	Folder labels and frozen RGB pseudo boxes/masks	Query boxes; dense-mask and global score heads	Unified three-seed localization
WS-DA-DETR	Folder labels and RGB-derived pseudo targets	Query boxes, dense mask, image class, health, and condition score	Recognition, localization, and condition scoring

**Table 9:** Configurations of the external query-based comparators. Shared heads, task losses, and learning rates are defined below; backbone and pretraining differences are retained.

Comparator	Backbone and Initialization	Added Output Heads	Training Configuration
Weak DETR	ResNet-50 + FPN; pretrained DETR	Shared $P_{3}$ mask and linear score heads	Shared task losses and learning rates
Weak Deformable DETR + DN	ResNet-50; multi-scale deformable attention; official weights	Shared $P_{3}$ mask and linear score heads	Shared task settings; DN-DETR denoising
Weak DINO	ResNet-50; official DINO weights	Shared $P_{3}$ mask and linear score heads	Shared task losses and learning rates
Weak RT-DETR	ResNet-50; official RT-DETR weights	Shared $P_{3}$ mask and linear score heads	Shared task losses and learning rates
WFS-DETR	Large-scale pretraining not matched	Not evaluated	Not directly comparable
Omni-DETR	Teacher–student and mixed annotations not matched	Not evaluated	Not directly comparable

**Table 10:** Recorded source repositories and implementation identifiers for the four evaluated query-based comparators.

Comparator	Source Repository	Recorded Initialization or Configuration
Weak DETR	facebookresearch/detr	Commit 4e1a3f9; checkpoint detr-r50-e632da11.pth
Weak Deformable DETR + DN	fundamentalvision/Deformable-DETR	DN-DETR denoising: 100 groups, noise scale 0.4, coefficient 1.0
Weak DINO	IDEA-Research/detrex	ResNet-50; official DINO weights
Weak RT-DETR	PaddlePaddle/PaddleDetection	ResNet-50; rtdetr_r50vd_6x_coco.yml

**Table 11:** Mixed-domain image-level recognition results. The five reference architectures provide classification baselines. Values are mean ± standard deviation over seeds 42, 3407, and 2026.

Model	Accuracy (%)	Macro-Precision (%)	Macro-Recall (%)	Macro-F1 (%)	Health AUC (%)
ResNet-50 [38]	83.5±0.8	81.2±1.0	80.5±0.9	80.8±0.9	91.2±0.5
EfficientNet-B3 [39]	85.4±1.1	83.8±1.0	83.0±1.1	83.4±1.0	93.0±0.6
ConvNeXt-Tiny [19]	87.6±0.7	85.9±0.8	85.5±0.7	85.7±0.7	94.8±0.4
ViT-B/16 [17]	86.9±1.0	85.1±1.1	84.7±1.0	84.9±1.0	94.2±0.5
Swin-T [18]	88.2±0.9	86.7±0.9	86.2±0.8	86.4±0.8	95.3±0.3
**WS-DA-DETR (ours)**	89.5±0.6	87.6±0.5	87.3±0.6	87.4±0.5	96.3±0.3

Boldface identifies the proposed model and the best value for each metric.

**Table 12:** Intrinsic pseudo-label quality and downstream localization on the same 200-image held-out manual subset. Pixel AUPR is reported on a 0–1 scale; the remaining measures are percentages.

Representation	Pixel AUPR	Mask IoU (%)	Box ${\mathrm{AP}}_{50}$ (%)	Recall@0.5 (%)
Raw anomaly map *A* and its frozen-threshold mask/box	0.41	28.5	31.2	52.8
Final pseudo mask and pseudo box	–	35.8	38.4	59.1
Weak DETR baseline after learning	–	36.5	42.1	62.5
**WS-DA-DETR after learning**	–	48.7	54.6	74.5

Boldface identifies the proposed model.

**Table 13:** Task-aligned box localization, dense-mask overlap, and localization recall on the 200-image manually annotated test subset. Values are mean ± standard deviation over three seeds.

Method	${\mathrm{AP}}_{50}$ (%)	${\mathrm{AP}}_{50:95}$ (%)	Mask IoU (%)	Recall@0.5 (%)
CAM map [13]	38.5±2.1	20.1±1.5	32.4±1.8	58.2±2.5
SEAM [21]	41.2±1.9	22.5±1.4	35.1±1.7	61.0±2.3
LCTR [15]	43.8±1.7	24.1±1.3	37.6±1.5	63.8±2.0
CATR [22]	46.1±1.6	25.8±1.2	40.2±1.4	66.4±1.9
WeCLIP [24]	44.5±1.8	24.6±1.3	38.9±1.5	64.7±2.1
Weak DETR [9]	42.1±1.8	22.8±1.3	36.5±1.6	62.5±2.2
Weak Deformable DETR, DN off	43.9±1.7	23.8±1.3	38.1±1.6	63.4±2.1
Weak Deformable DETR + DN [10]	45.3±1.6	25.0±1.2	39.8±1.5	65.1±2.0
Weak DINO [11]	48.4±1.5	27.3±1.0	42.2±1.4	68.5±1.8
Weak RT-DETR [12]	47.8±1.7	27.1±1.1	41.5±1.5	67.8±2.1
**WS-DA-DETR (ours)**	54.6±1.2	32.3±0.7	48.7±1.1	74.5±1.4
WFS-DETR [29]	Not evaluated: Unmatched pretraining			
Omni-DETR [30]	Not evaluated: Unmatched annotation regime			

Boldface identifies the proposed model and the best value for each metric.

**Table 14:** Human-rating reliability and agreement of the predicted visible surface-condition score with the mean human rating.

Statistic	Estimate	95% CI	Interpretation
Quadratic-weighted Cohen’s κ between raters	0.71	[0.64, 0.78]	Five-level ordinal agreement
ICC(2,1) between raters	0.74	[0.67, 0.80]	Absolute numerical-score agreement
Unmatched-aware mean box IoU	0.59	[0.52, 0.66]	Pre-adjudication spatial agreement
Unmatched-aware mean mask Dice	0.63	[0.56, 0.70]	Pre-adjudication spatial agreement
WS-DA-DETR versus mean rating, Pearson *r*	0.68±0.03	[0.61, 0.74]	Linear model–human association
WS-DA-DETR versus mean rating, Spearman ρ	0.66	[0.58, 0.73]	Rank model–human association
WS-DA-DETR versus mean rating, MAE	0.14	[0.12, 0.16]	Absolute error, 0–1 scale
WS-DA-DETR versus mean rating, RMSE	0.18	[0.16, 0.21]	Quadratic error, 0–1 scale

**Table 15:** Condition-score comparison on the same 200 images and paired human ratings. Pearson *r* is mean ± SD over three seeds; Spearman ρ, MAE, and RMSE use pooled held-out predictions.

System	Pearson *r*	Spearman ρ	MAE	RMSE
Weak DETR	0.54±0.04	0.51	0.19	0.24
Weak Deformable DETR + DN	0.57±0.04	0.54	0.18	0.23
Weak DINO	0.61±0.03	0.58	0.16	0.21
Weak RT-DETR	0.59±0.04	0.56	0.17	0.22
WS-DA-DETR	0.68±0.03	0.66	0.14	0.18

**Table 16:** Individual runs on the same 200-image manual subset. Percentage units apply to all spatial metrics.

Seed	Images	${\mathrm{AP}}_{50}$	${\mathrm{AP}}_{50:95}$	Mask IoU	Recall@0.5	Pearson *r*
42	200	55.9	33.1	49.9	76.1	0.71
3407	200	54.4	32.0	48.5	74.2	0.67
2026	200	53.5	31.8	47.7	73.3	0.66
Mean ± SD	200	54.6±1.2	32.3±0.7	48.7±1.1	74.5±1.4	0.68±0.03

**Table 17:** Class-and domain-specific localization. Lesion counts are recall denominators; each partition sums to 318 lesions. Spatial metrics are percentages.

Subset	Images	Lesions	${\mathrm{AP}}_{50}$	${\mathrm{AP}}_{50:95}$	Mask IoU	Recall@0.5
Bruise	65	77	52.1	30.4	46.8	71.4
Rot	80	113	58.7	35.1	52.4	78.8
Scab	55	128	51.3	29.8	45.6	72.7
Real	130	215	52.8	30.9	47.1	72.1
Synthetic	70	103	57.9	34.8	51.6	79.6

**Table 18:** Paired performance differences with image-bootstrap 95% confidence intervals. Spatial differences are percentage points.

Comparison	Metric	Mean Difference	Paired 95% CI
WS-DA-DETR minus Weak DETR	${\mathrm{AP}}_{50:95}$ (points)	+9.5	[7.8, 11.2]
WS-DA-DETR minus Weak DETR	Mask IoU (points)	+12.2	[10.1, 14.3]
WS-DA-DETR minus Weak DETR	Recall@0.5 (points)	+12.0	[9.6, 14.4]
WS-DA-DETR minus final pseudo mask	Mask IoU (points)	+12.9	[10.7, 15.1]
WS-DA-DETR minus final pseudo box	${\mathrm{AP}}_{50}$ (points)	+16.2	[13.9, 18.5]
WS-DA-DETR minus pseudo score	Pearson *r*	+0.19	[0.12, 0.26]

**Table 19:** Factorial and sequential ablation under the mixed-domain protocol. The four detector rows beginning with the internal multi-task weak DETR baseline isolate SDTE, DGQR, and their combination; subsequent rows add auxiliary regularization. Values are mean ± standard deviation over three seeds.

Variant	Health Macro-F1 (%)	${\mathrm{AP}}_{50:95}$ (%)	Surface-Condition *r*
Classification only	95.0±0.5	–	–
Internal multi-task weak DETR baseline	95.5±0.4	25.5±1.2	0.52±0.04
+SDTE only	96.0±0.3	28.2±1.0	0.58±0.03
+DGQR only	95.9±0.4	27.5±1.1	0.56±0.04
+SDTE+DGQR	96.3±0.3	31.0±0.9	0.63±0.03
+SDTE+DGQR+contrastive	96.5±0.3	31.8±0.8	0.66±0.03
**Full WS-DA-DETR (+consistency)**	96.7±0.2	32.3±0.7	0.68±0.03

Boldface identifies the full proposed model and the best value for each metric.

**Table 20:** Cross-domain generalization. “S→R” trains/validates on synthetic images and tests on all real images; “R→S” reverses the domains.

Model	Protocol	Accuracy (%)	Macro-F1 (%)	Health AUC (%)
ConvNeXt-Tiny	S→R	72.5±1.5	70.0±1.8	85.0±1.2
WS-DA-DETR	S→R	78.3±1.2	76.5±1.4	90.2±1.0
ConvNeXt-Tiny	R→S	84.0±1.0	82.2±1.2	93.5±0.8
WS-DA-DETR	R→S	86.8±0.9	85.5±1.0	95.5±0.6

Boldface indicates the best value within each transfer protocol.

**Table 21:** Reported model complexity and model-only inference efficiency at 384×384 on an NVIDIA RTX 3090. Latency is a batch-one aggregate after warm-up.

Model	Parameters	MACs	Latency (ms)	FPS
ConvNeXt-Tiny classifier	28.6 M	4.5 G	12.1 ms	82.6
DETR baseline	37.2 M	8.2 G	24.8 ms	40.3
**WS-DA-DETR**	41.4 M	9.5 G	30.2 ms	33.1

Boldface identifies the proposed model.

## Data Availability

The source images and folder labels are publicly available as *Healthy and Defective Fruits*, version 1, on Kaggle [32], at https://www.kaggle.com/datasets/luischuquimarca/healthy-and-defective-fruits (accessed on 27 September 2026). Only the 10,000-image apple subset was used. Seed-42 mixed-domain and transfer manifests are retained in the project. Study-generated COCO annotations for 200 images and 318 lesions, paired human ratings, per-seed predictions, bootstrap outputs, run logs, and model checkpoints have been recovered and will be uploaded through the journal submission system. These records are distinct from the publicly distributed images.
